# Process Optimization and Performance Evaluation of TSV Arrays for High Voltage Application

**DOI:** 10.3390/mi14010102

**Published:** 2022-12-30

**Authors:** Liuhaodong Feng, Shuwen Zeng, Yongquan Su, Lihao Wang, Yang Xu, Song Guo, Shuo Chen, Yucheng Ji, Xinlin Peng, Zhenyu Wu, Shinan Wang

**Affiliations:** 1 School of Microelectronics, Shanghai University, Shanghai 201800, China; 2 Shanghai Industrial μTechnology Research Institute, Shanghai 201800, China; 3 Shanghai Institute of Microsystem and Information Technology, Chinese Academy of Sciences, Shanghai 201800, China

**Keywords:** TSV, DRIE, hole sidewall smoothing, bottom-up electroplating, dielectric performance evaluation

## Abstract

In order to obtain high-quality through-silicon via (TSV) arrays for high voltage applications, we optimized the fabrication processes of the Si holes, evaluated the dielectric layers, carried out hole filling by Cu plating, and detected the final structure and electric properties of the TSVs. The Si through-hole array was fabricated in an 8-inch Si substrate as follows: First, a blind Si hole array was formed by the Si deep reactive etching (DRIE) technique using the Bosch process, but with the largest width of the top scallops reduced to 540 nm and the largest notch elimidiameternated by backside grinding, which also opens the bottom ends of the Si blind holes and forms 500-μm-deep Si through holes. Then, the sidewalls of the Si holes were further smoothed by a combination of thermal oxidation and wet etching of the thermal oxide. The insulating capability of the dielectric layers was evaluated prior to metal filling by using a test kit. The metal filling of the through holes was carried out by bottom-up Cu electroplating and followed by annealing at 300 °C for 1 h to release the electroplating stress and to prevent possible large metal thermal expansion in subsequent high-temperature processes. The TSV arrays with different hole diameters and spacing were detected: no visible defects or structure peeling was found by scanning electron microscopy (SEM) observations, and no detectable interdiffusion between Cu and the dielectric layers was detected by energy dispersive X-ray (EDX) analyses. Electric tests indicated that the leakage currents between two adjacent TSVs were as low as 6.80 × 10^−10^ A when a DC voltage was ramped up from 0 to 350 V, and 2.86 × 10^−9^ A after a DC voltage was kept at 100 V for 200 s.

## 1. Introduction

With the development of the microfabrication process, the integration of micro-electromechanical systems (MEMS) and integrated circuit (ICs) devices have been developing quickly, whereas the feature sizes have recently shrunk recently. However, according to Moore’s laws, the device feature sizes are approaching their physical limits. To improve their integrity, multilayer chip stacking techniques are coming into view. Through-silicon via (TSV) is a practical technology to vertically interconnect multiple chips. Usually, a TSV substrate is fabricated mainly by etching through holes in a silicon substrate, and by filling the holes with a conductive material such as Cu, W, and doped polysilicon. In the IC field, TSVs can be used for complementary metal-oxide semiconductor (CMOS) image sensors, SiGe power amplifiers, 3D stacked memory devices, and field-programmable gate array (FPGA) chip integration [1,2,3]. The application of TSVs in the IC field can reduce the size of devices, improve signal transmission, and can even address the manufacturing challenges of large chips. In the MEMS field, there are also advantages of TSVs in vacuum packaging for inertial sensors including gyroscopes and accelerometers [4], in 3D stacking of sensors and driver circuits to enable high performance, as well as miniaturization of devices [5]. TSVs can also reduce substrate bonding difficulties while keeping the bonding strength, and they can increase the density of electrodes.

Some electrostatically driven MEMS devices require high input voltages. A scratch drive actuators (SDAs) system is driven by pulsed electrostatic forces generated by the input pulse voltage up to 80 V [6]. Shuaibu, A.H. et al. proposed a DC switch, on which a driven voltage of up to 350 V is required [7]. If the normal TSV is applied to such devices, there will be a risk of breakdown. The two main modes of TSV failure are dielectric breakdown and electrical migration. A rupture in the barrier layer or the dielectric layer facilitates the filling metal to diffuse into the silicon substrate. Such a process will diminish the electrical performance of a TSV.

In some applications, TSVs need to be formed on thick Si substrates (e.g., substrate thickness ≥ 400 μm) to meet the requirements of high mechanical strength not only for final usage but also for the fabrication processes. The thicker the Si substrate, however, the more difficult the TSV fabrication process, from hole formation to dielectric layer deposition to metal filling.

The through Si holes for TSVs are often realized by the famous Bosch etching process due to the high fabrication rate and controllable high aspect ratio hole geometry. The Bosch process is characterized by alternating the etching and passivating steps during the multiple-cycle structure fabrication, which naturally leaves a periodic scallop on the sidewall of a hole. The scallops may cause stress concentration, which results in the cracking of the dielectric and barrier layers deposited on the sidewall [8]. The stress may be enhanced in a subsequent thermal process, especially when the edges of the scallop are sharp. The scallops also increase the difficulty of conformal deposition of dielectric/barrier/seed layer on the hole sidewall due to the shadowing effect, resulting in possible defects such as voids in the metal filling, junction of the metal and sidewalls, and spalling of Cu in the chemical-mechanical planarization (CMP) after electroplating [9,10]. It is then desired to reduce the size of the scallop to a reasonable degree in the TSV application. The geometry of the hole openings also needs to be optimized since a bad corner shape is prone to dielectric breakdown due to stress concentration [11]. In the case that through holes of different diameters are necessary for the same Si substrate, the micro-loading effect [12] in the DRIE usually results in an etch rate difference between the holes: the smaller the hole opening, the lower its etch rate and vice versa. However, the problem lies in that in order to completely open the hole of the smallest opening, the hole of the largest opening must be over-etched, causing its bottom opening to undesirably expand. MS Gerlt, et al. managed to etch trenches of various widths within a depth difference of less than 1.5% by adjusting the ratio between the duration of the passivation process and the etching process in the DRIE [13]. However, the problem of narrow process windows emerges. “Notching” is usually a bothersome effect in DRIE, especially in through-hole fabrication due to charge accumulation on the hole bottom which deflects the arriving ions and causes lateral etching of the bottom sidewall. The notches may lead to incomplete coverage of sequent liner oxide and Cu seed [14]. Kim et al. realized “notch-free” DRIE by depositing metal on the backside of the substrate to release the charges [15]. However, the metal layer on the backside will cause metal ion contamination in the subsequent high-temperature oxidation process. Therefore, such a method is not suitable for our TSV formation.

The interlayer stress also needs to be examined in TSVs since the coefficients of thermal expansion (CTE) and other mechanical parameters of the related materials vary greatly as shown in Table 1 [16].

How to prevent the metal (e.g., Cu) diffusion into the dielectric layer is another issue that can affect the insulation performance of TSVs. The energy dispersive X-ray (EDX) analysis is suitable for detecting the diffusion of Cu ions into the SiO_2_ dielectric layer [17]. The migration of metal atoms, which can cause void formation at the via interface when an electric current is applied [18,19,20], shall be avoided as well. 

This work investigates and optimizes the silicon through-via fabrication process for high-voltage applications. Then, the dielectric properties were evaluated before and after electroplating.

**Table 1:** Material properties at 25 °C [21,22,23].

Material Parameters	Si	SiO_2_	Si_3_N_4_	Ti	Ta	Cu
CTE (−40 °C to 125 °C) (ppm/°C)	2.6	0.94	2.8	8–10	6.5	17.7
Young’s modulus (GPa)	130.91	70	~220	116	186	104.2
Poisson’s ratio	0.28	0.17	0.26	0.34	0.35	0.35
Yield strength (MPa)	120			140	170	70
Ultimate strength (MPa)			~172	220	450	220

## 2. Methods

In his work, TSVs have been fabricated in 8-inch Si substrates thicker than 400 μm. The substrates are p-type with a resistivity between 1 and 100 Ω-cm. TSV arrays with different hole opening sizes and via spacing are arranged on the substrate with the layout combination shown in Table 2.

The Si through holes were fabricated by DRIE using the Bosch process on a SPTS Omega C2L Rapier machine. The time of de-passivation etching (E1) is 1 s, and the time of Si etching (E2) is 8 s. For E2, the source powers were set to 3000 W for the center coil and 1000 W for the outer coil. The bias power was increased from 470 W to 500 W, whereas the SF_6_ gas flow rate was 650 sccm. The de-passivation gas was also SF_6_ with gas flow rates of 300 sccm for the center pipeline and 50 sccm for the outer pipeline. We first tried to obtain the through holes by etching through the substrate directly. However, we found it difficult to avoid large notches at the bottom openings of the holes by optimizing the DRIE recipe. Then, we replaced the through-via etching with a combination of blind-via etching and backside thinning. That is, we first formed blind holes in the substrate with all the holes deeper than desired; then, we carried out backside mechanical grinding by a grinder (ACCRETECH, HRG 300) to open the bottom ends of the holes and at the same time to thin the substrate to the desired thickness. By this method, even if notches have occurred at the hole bottoms, they can be removed by backside grinding. As a result, “notch free” through holes can be obtained relatively simply. By using the grinder’s probe to monitor the thickness of the Si substrate in real-time, we controlled the thickness of the substrate with an accuracy of no more than 2 μm and maintained the parallelism of the top and bottom surfaces of the substrate.

Since it is difficult to reduce the sidewall scallops by fine-tuning the DRIE recipe, after the hole DRIE, we adopted the sidewall smoothing process where thermal oxidation was performed followed by wet etching of the oxide layer. We adjusted the thickness of the thermal oxide so that the most protruding parts of the scallops can be completely oxidated and then removed via the smoothing process.

**Figure 1:**
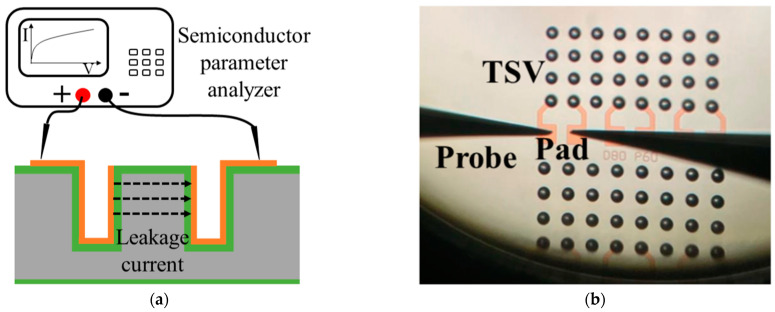
TSV “pre-plating” leakage current test. (**a**) schematics of the test kit, (**b**) microscopy of the testing probes on the pads of two adjacent TSVs.

We then formed dielectric layers of different materials and different thicknesses on the surface of the substrate and on the through hole sidewalls. We evaluated their insulating capability with the test kit shown in Figure 1a. For the insulating capability test, a TiN film was deposited by physical vapor deposition (PVD) on the dielectric layer and was patterned on the substrate surface as metal electrodes (also called pads). The leakage current between the sidewalls of two adjacent holes was measured by applying a DC voltage to the electrodes shown in Figure 1b. Time-dependent dielectric breakdown (TDDB) tests were also conducted to confirm the insulating capabilities of the dielectric films. In order to confirm the sidewall coverage of the TiN film, the leakage current of a silicon blind with a diameter of 80 μm and a depth of 512 μm was first detected. Afterward, the insulating capability of a 500 nm thick TEOS-PECVD (PECVD: plasma-enhanced chemical vapor deposition) silicon oxide and a 500 nm thick thermal silicon oxide were tested. Then, a 2 μm thick silicon oxide dielectric layer using a high-temperature thermal oxidation process was applied to achieve superior dielectric performance. 

In order to achieve void-free metal filling, we adopted the bottom-up Cu electroplating method, which is widely used in high quality TSV filling [24,25,26,27]. An annealing process was carried out at 350 °C for 1 h after Cu plating to release the plating stress and, most importantly, to avoid possible irreversible Cu deformation in subsequent high-temperature processes [28]. Before metal filling, a thin, 20 nm thick Ti film was sputtered as the barrier layer with a sputtered Cu thin film as the seed layer. The bottom-up Cu electroplating was carried out until Cu pillars protruded completely from the top side of all the holes. Then, wet etches were conducted to remove the electroplated Cu layer and the Cu seed layer, as well as the Ti film, on the surfaces of the Si substrate. Through all these processes, we achieved individual Cu vias filled in the Si through holes.

Finally, we obtained the cross-sectional views of TSVs with a diameter of 80 μm and depth of 500 μm by focused ion beam (FIB) cutting and scanning electron microscopy (SEM) observations. Energy dispersive X-ray (EDX) analyses were also carried out to confirm whether or not there was Cu diffusion into the dielectric layers. 

## 3. Results

### 3.1. DRIE

**Figure 2:**
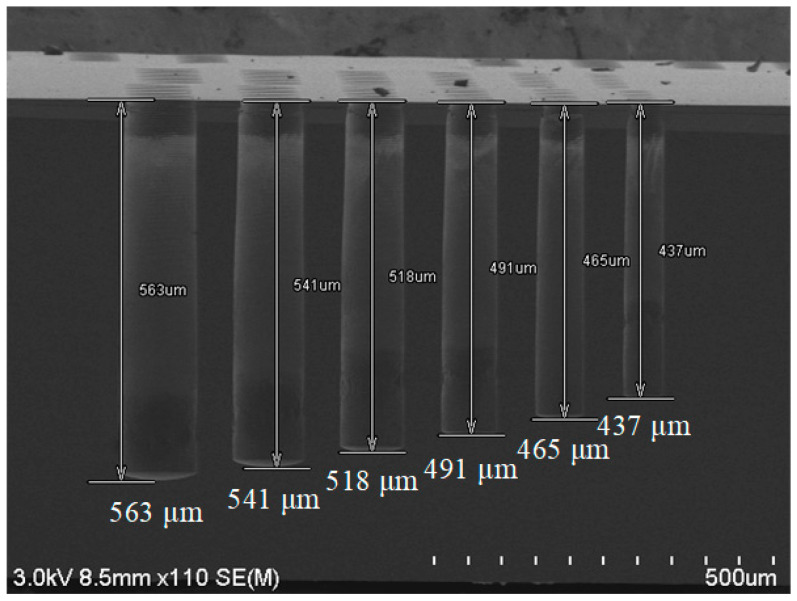
SEM image of Si holes of different diameters. Depth = 563 μm @diameter = 100 μm, depth = 437 μm @diameter = 50 μm.

**Figure 3:**
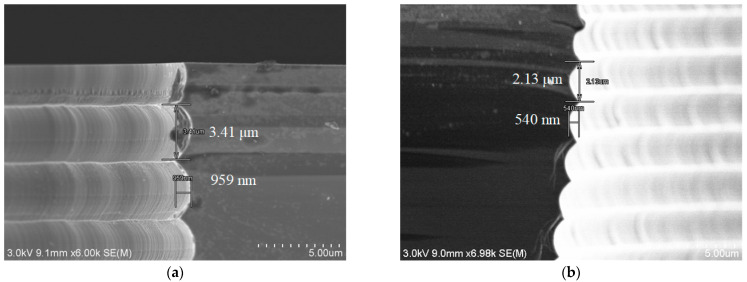
Scallops on the top sidewalls of holes with an opening of 80 μm: (**a**) Before optimizing the DRIE parameters. The largest width and depth of the scallop is 3.41 μm and 959 nm, respectively; (**b**) After optimizing the DRIE parameters. The largest width and depth of the scallop becomes 2.13 μm and 540 nm, respectively.

First, experiments were carried out to reduce the scallop size. As shown in Figure 2, blind hole arrays of different hole diameters from 50 μm to 100 μm were formed by DRIE in an 8-inch silicon wafer 725 μm thick. The overall morphology is slightly reverse-tapering. The micro-loading effect is obvious: the largest 100-μm-diameter hole has the largest depth of 563 μm, and the smallest 50-μm-wide hole has the smallest depth of 437 μm, whereas other holes with diameters in between have the following depths. In Figure 3, the sidewall of the 80-μm-wide hole shown in Figure 2 is enlarged in order to have a better angle of observation at the scallops. Figure 3a shows the scallop on the sidewall near the top opening of the hole. The scallop has a width of 3.41 μm and a depth of 959 nm. Such a large scallop is difficult to eliminate by a smoothing process. Therefore, the etching process was optimized. First, the etch time of a single step during the process of etching the top part of the hole is shortened to 0.9 s for E1 and 6 s for E2 so that the size of the scallop is reduced. In order to decrease the negative impact from the shortening of etch time, the overall hole shape, as well as the bottom roughness, the flow rate of the etching gas SF_6_ was ramped up from 650 sccm to 850 sccm through the entire hole etching process. Meanwhile, the time of E1 and E2 were gradually increased to 1.5 s and 10 s, respectively. To reduce lateral etching from the middle part of the hole, the reaction gas for the etching process of the passivation layer was changed from SF_6_ to O_2_, since O_2_ has a higher etching selectivity to silicon than SF_6_. O_2_ was used to remove the passivation layer effectively while reducing the damage to the silicon sidewall as compared to SF_6_. The gas flow of O_2_ for E1 was increased from 100 sccm to 125 sccm. The source powers of E2 were also decreased from 3000/1000 W to 3000/750 W to reduce lateral etching. The bias power was increased to 560 W to avoid etch stop at the bottom of the hole. We also increased the cycle number to compensate for the reduced etch rate caused by the replacement of the de-passivation etching gas. By the above tuning of the DRIE recipe, the largest scallop size was decreased to 2.13 μm in width and 540 nm in depth, with a depth decrease of 43.7%.

**Figure 4:**
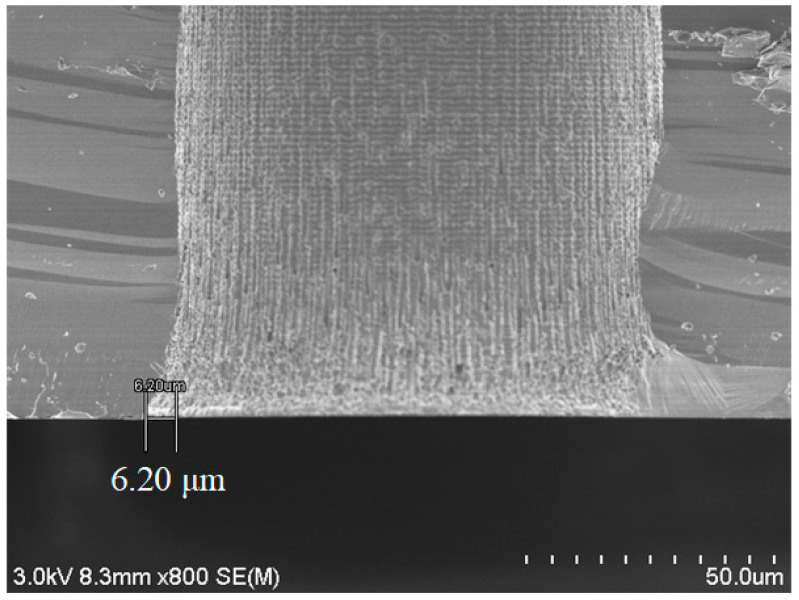
SEM image of the bottom notch of a through hole after DRIE.

Secondly, the notch problem was studied. As shown in Figure 4, when a through hole was formed directly by DRIE in a 500 μm thick silicon substrate, a notch as wide as 6.2 μm is observed when the hole diameter is 80 μm. As mentioned earlier, the larger the hole diameter, the larger the notch size. In the meantime, the sidewall close to the bottom opening is quite rough. However, in all through holes with diameter from 50 μm to 100 μm, these problems were not observed when the holes were fabricated by the combination of blind via etching and backside thinning with a starting substrate 725 μm thick.

### 3.2. Sidewall Smoothing

**Figure 5:**
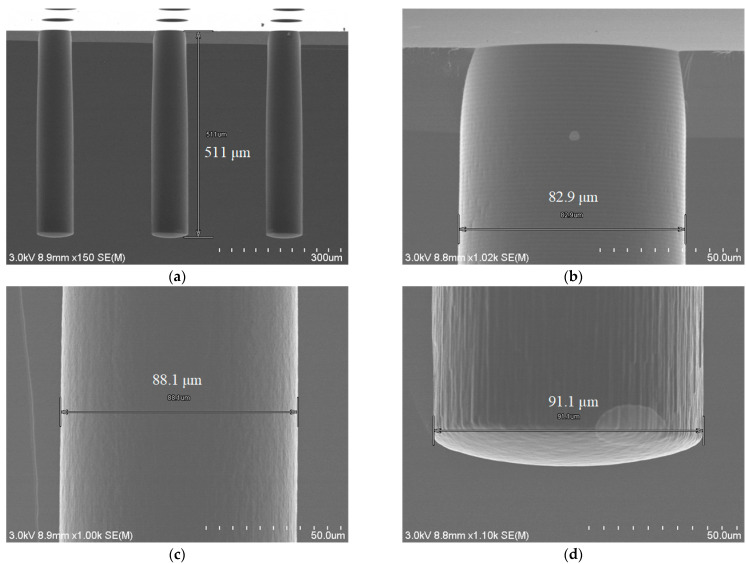
Hole profiles after sidewall smoothing by thermal oxide layer formation and removal: (**a**) Profile of the entire holes, (**b**) top profile, (**c**) middle profile, (**d**) bottom profile.

As discussed Section 3.2, the largest depth of scallop is as large as 500 nm despite the optimization of the DRIE process. Figure 5 shows the hole profiles resulted from sidewall smoothing. The results are obtained as follows: blind holes of 500 μm depth were first formed by optimized DRIE, and then a silicon oxide sacrificial layer 2 μm thick was formed on the hole sidewalls by thermal oxidation at 1100 °C. The silicon oxide layer was completely removed by using buffered oxide etch (BOE) and dilute hydrofluoric acid (DHF) etch. BOE was found effective in removing the oxide layer on the sidewall from the top to the middle of the hole; however, the oxide layer was left on the bottom sidewall almost intact. On the other hand, DHF, although slow in etch rate, was found effective in removing the bottom oxide layer. This happened because DHF has better wetting effect than BOE and was able to enter the bottom of the blind hole easily. As shown in Figure 5, after smoothing, the hole sidewall got as smooth as needed: only fine traces of the scallop are left in the top part while there are no longer visible scallops in the middle and bottom parts.

### 3.3. “Pre-Plating” Dielectric Property Test

**Figure 6:**
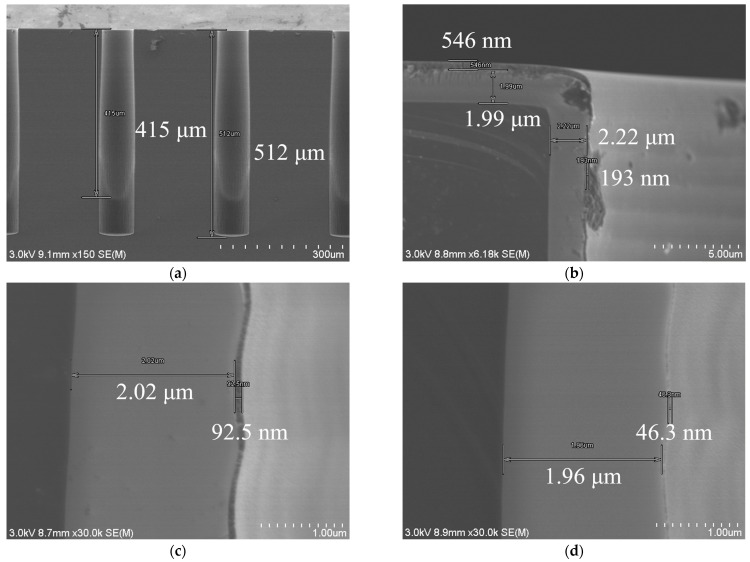
SEM images of TiN thin films deposited by PVD: (**a**) The film reaches a depth up to of 415 μm in the blind holes 80 μm in diameter, (**b**) TiN thin-films on the substrate surface (546 nm in thickness) and on the top sidewall (193 nm in thickness), (**c**) TiN thin-film on the middle sidewall, with a thickness of 92 nm, (**d**) TiN thin-film on middle-bottom sidewall, with a thickness of 46 nm.

First, as the electrical test electrodes, TiN films were formed by PVD on the substrate surface. The hole sidewalls and the film coverage was confirmed. Figure 6 shows the result of TiN film coverage for blind holes of depth > 400 μm. According to a TiN film thickness of 546 nm on the surface of the substrate, the thickness on the top sidewall is 193 nm, indicating a coverage rate of 35.3%. The TiN film thickness drops to 92 nm on the middle sidewall, and drops to 46 nm on the middle bottom sidewall. The TiN film was no longer visible when the sidewalls were deeper than 415 μm. Based on this, we conducted the “pre-plating” dielectric property test in blind holes 300 μm in depth, where high-enough TiN film coverage can be ensured on both the hole sidewalls and bottoms.

#### 3.3.1. Hole Diameter Dependence of the Dielectric Property in Holes with Sidewalls Unsmoothed

**Figure 7:**
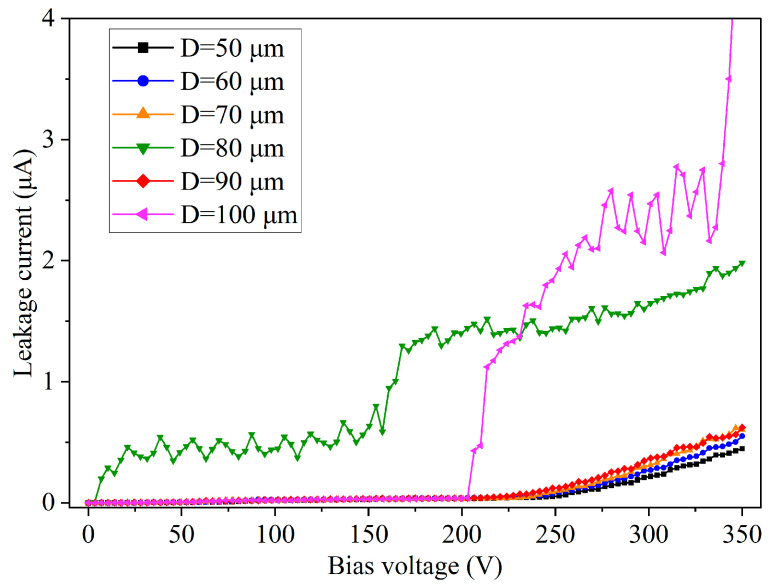
Leakage current test results of PECVD oxide film (500 nm thick on the substrate surface) formed in Si blind holes of diameters from 50 μm to 100 μm, spacing of 60 μm, where the hole sidewalls were not smoothed.

Figure 7 shows the hole diameter dependence of the dielectric property obtained in 300-μm-deep blind holes without sidewall smoothing. On the hole sidewalls, a silicon oxide film was deposited by using TEOS-PECVD, and then a TiN film was deposited and patterned as the electrodes. The thicknesses of the oxide and TiN films are both 500 nm on the substrate surface. As seen in Figure 7, when DC voltage was applied from 0 V to 350 V, the leakage currents increased; the larger the hole diameter, the higher the leakage current. The dielectric film breakdown occurred at a voltage of about 330 V in the hole with the largest diameter (100 μm). This diameter dependence may originate from worse oxide film coverage in larger holes, since larger holes have larger depth (as seen in Figure 2), which lead to thinner film formation on the bottom sidewalls, especially at the bottom corners of the blind holes. Another important factor may be the scallop. As stated by Hsin et al., due to the nature of the Bosch process, the larger the diameter of holes, the larger the size of sidewall scallop [29]. It is easy to understand that a larger scallop indicates a worse dielectric film coverage in the scallop valleys due to a stronger shadowing effect. The abnormally high leakage current in the 80 μm diameter hole, however, may result from defects in the silicon oxide film caused by uncertain factors.

#### 3.3.2. Hole Spacing Dependence of Dielectric Properties in Holes with Unsmoothed and Smoothed Sidewalls

**Figure 8:**
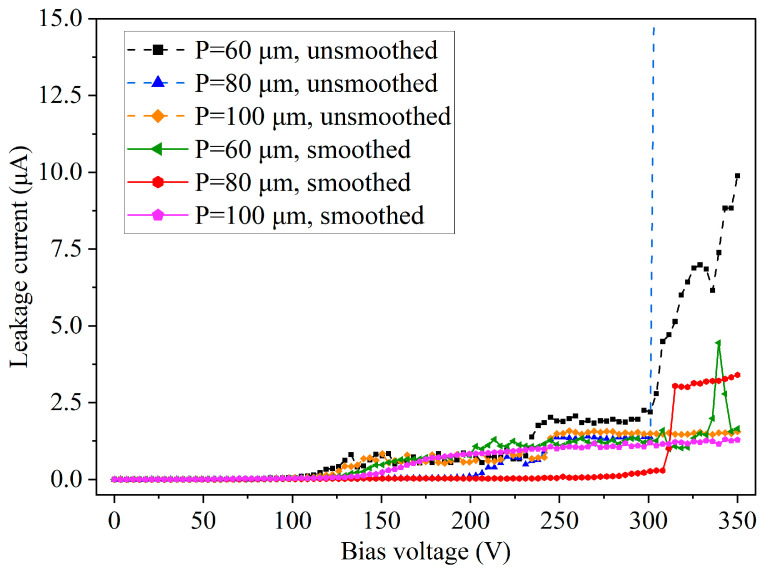
Leakage current test results of PECVD oxide film (500 nm thick on the substrate surface) formed in Si blind holes 80 μm in diameter with various hole spacing (60 μm, 80 μm, and 100 μm), where some of the hole sidewalls were smoothed and some unsmoothed.

Figure 8 shows the hole spacing dependence of dielectric properties in holes with unsmoothed and smoothed sidewalls. For both the sidewall unsmoothed and smoothed holes, the Si blind holes have a diameter of 80 μm, whereas the hole spacing varies from 60 μm to 80 μm to 100 μm. The PECVD silicon oxide film and the TiN film both have a thickness of 500 nm on the substrate surface, as shown in Section 3.3.1. The dielectric films in the holes with unsmoothed sidewalls indicate larger leakage currents than those in holes with smoothed sidewalls. In holes with unsmoothed sidewalls and a spacing of 80 μm, the dielectric films show a breakdown at 300 V. The above results indicate again that the sidewall scallop is an important cause of dielectric breakdown, while the sidewall smoothing process has significantly improved the insulating performance of the TSVs. The hole spacing, however, did not show definite effect on the dielectric properties when its size varied from 60 μm to 100 μm.

#### 3.3.3. Dielectric Properties of TEOS-PECVD and Thermal Silicon Oxide Films

**Figure 9:**
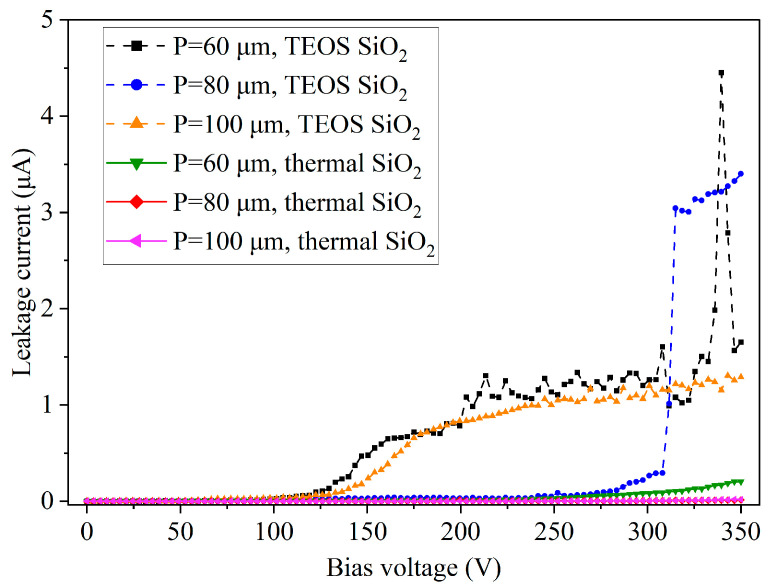
Leakage current test results of PECVD and thermal oxide films (both 500 nm thick on the substrate surface) formed in Si blind holes 80 μm in diameter with various hole spacing (60 μm, 80 μm, and 100 μm), where the hole sidewalls were smoothed.

Figure 9 shows the comparison of the dielectric properties for TEOS-PECVD and thermal silicon oxide films, both having a film thickness of 500 nm on the substrate surface. The Si blind holes 80 μm in diameter with various hole spacing (60 μm, 80 μm, and 100 μm) have their sidewalls smoothed. Obviously, the dielectric property of the thermal silicon oxide films is superior to that of TEOS-PECVD films. The TEOS-PECVD silicon oxide film shows a breakdown near 310 V in holes with a spacing of 80 μm, whereas all the thermal silicon oxide films still maintain a low leakage current even when the DC voltage is raised to 350 V. 

### 3.4. Metal Filling

**Figure 10:**
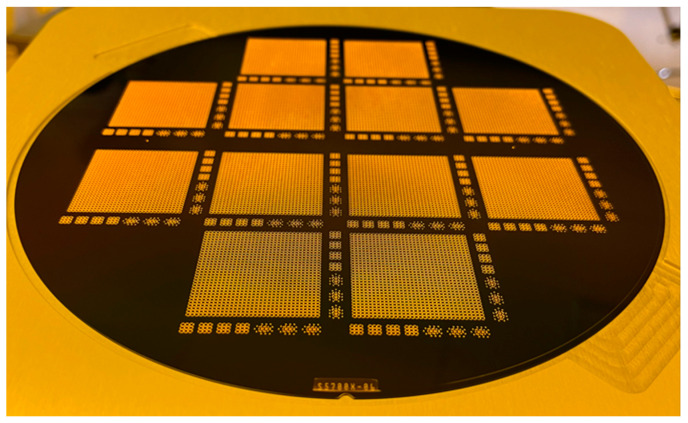
Photograph of TSV arrays after Cu electroplating in an 8-inch Si substrate of 500 μm in thickness.

**Figure 11:**
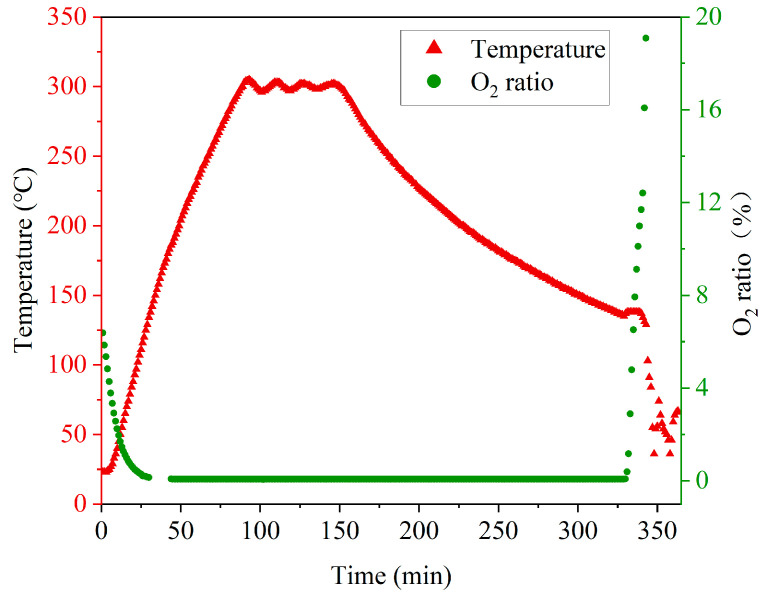
Temperature curve and the O_2_ ratio in the oven during the annealing after Cu electroplating.

Based on the experimental results described in Section 3.3, through hole arrays were fabricated with a combination of optimized DRIE and backside thinning, followed by a smoothing process and a 2-μm-thick thermal silicon oxide formation. The resulting substrates have a thickness of 500 μm, and the holes have diameters ranging from 50 μm to 100 μm. The spacing of the through holes ranges from 60 μm to 100 μm. Figure 10 is a photograph of TSV arrays after Cu electroplating in an 8-inch Si substrate 500 μm thick. After Cu plating, an annealing process was conducted at 300 °C for 1 h in an oven under a nitrogen atmosphere. Figure 11 shows the measured temperature curve and the O_2_ ratio in the atmosphere during the annealing process. After annealing, wet etch was carried out to remove the metal layers on the substrate surfaces. 

**Figure 12:**
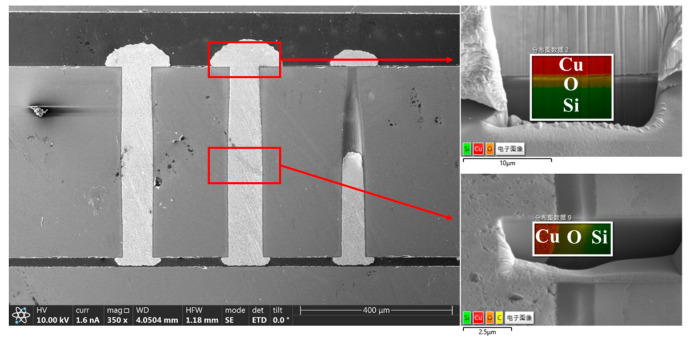
Cross-sectional view of the TSVs after annealing with EDX analysis results.

Figure 12 shows the cross-sectional view of the TSVs after annealing together with EDX analysis results. The TSVs have a diameter of 80 μm and a thickness of 500 μm. From the polished cross sections, no void or defects were observed. According to the EDX analyses, no diffusion of Cu into the dielectric layer has occurred. 

Dielectric property tests also provide satisfactory results. When the probes for electric detection were placed directly on the protruded Cu heads of two adjacent TSVs (80 μm in diameter, 90 μm in spacing), the leakage current is as small as 6.80 × 10^−10^ A at a DC voltage of 350 V. In the TDDB test, the leakage current between adjacent TSVs (80 μm in diameter, 90 μm in spacing) is approximately 2.86 × 10^−9^ A after a DC voltage of 100 V being continuously applied for 200 s. No breakdown phenomenon happened during the voltage increase and the TDDB tests.

## 4. Conclusions

Fabrication processes for TSV arrays aimed at applications at high voltage were studied systematically. TSVs in need have been obtained in 8-inch silicon substrates with a maximum thickness 500 μm, whereas the hole diameters vary from 50 μm to 100 μm, and the hole spacing varies from 60 μm to 100 μm. An important factor was found to worsen the dielectric property of the TSVs during hole DRIE. By optimizing the DRIE recipe and then performing a smoothing process combined with thermal oxidation and wet etching of the oxide layer, the scallops were almost eliminated. The notch structures, which often occurred at the bottom opening of through holes by DRIE, were completely avoided by forming the through holes together with a combination of blind hole DRIE and backside grinding processes. As for the dielectric material, the thermal silicon oxide is found superior to the TEOS oxide. Defect-free metal filling was achieved by adopting the bottom-up Cu electroplating method. The fine structures, as well as their high dielectric performance of the obtained TSVs, indicate the usefulness of the developed TSV fabrication process.

## Figures and Tables

**Table 2:** Layout combination of TSV arrays.

Array No.	Diameter (μm)	Spacing (μm)
1	50	60, 70, 80, 90
2	60	60, 70, 80, 90
3	70	60, 70, 80, 90
4	80	60, 70, 80, 90, 100
5	90	60, 70, 80, 90
6	100	60, 70, 80, 90

## Data Availability

Not applicable.

## References

[1] Benkechkache A., Latreche S., Ghoualmi L. (2022). Overview and study of the 3D-TSV interconnects induced coupling in CMOS circuits. arXiv.

[2] Venkatesha S., Parthasarathi R. (2022). A Survey of fault models and fault tolerance methods for 2D bus-based multi-core systems and TSV based 3D NOC many-core systems. arXiv.

[3] Zhou J., Chen Y., Jing Y., Zhou P. (2022). The study of TSV-induced and strained silicon-enhanced stress in 3D-IC. Integration.

[4] Yeh Y.M., Chang S.J., Wang P.H., Hsueh T.J.A. (2022). TSV-Structured Room Temperature p-Type TiO2 Nitric Oxide Gas Sensor. Appl. Sci..

[5] Hirama I. New MEMS sensor process by TSV technology for smaller packaginge. Proceedings of the 2015 International Conference on Electronics Packaging and iMAPS All Asia Conference.

[6] Akiyama T., Collard D., Fujita H. (1997). Scratch drive actuator with mechanical links for self-assembly of three-dimensional MEMS. J. Microelectromech. Syst..

[7] Shuaibu A.H., Nabki F., Blaquière Y. A MEMS electrothermal actuator designed for a DC switch aimed at power switching applications and high voltage resilience. Proceedings of the 2022 20th IEEE Interregional NEWCAS Conference.

[8] Fan Z., Chen X., Wang Y., Jiang Y. (2022). Experimental research on performance degradation of TSV microstructure under thermal cycling, vibration and electrical stress. Microelectron. Reliab..

[9] Zhang J., Bloomfield M.O., Lu J.Q., Gutmann R.G., Cale T.S. (2005). Thermal stresses in 3D IC inter-wafer interconnects. Microelectron. Eng..

[10] Shingubara S., Shimizu T., Matsui K., Miyake Y., Torinari Y., Motoyoshi M., Watariguchi S., Watanabe H. TSV fabrication technology using direct electroplating of Cu on the electroless plated barrier metal. Proceedings of the 2022 IEEE International Interconnect Technology Conference.

[11] Ranganathan N., Lee D.Y., Youhe L., Guoqiang L., Krishnamachar P., Leongpey K. (2011). Influence of Bosch etch process on electrical isolation of TSV structures. Trans. Compon. Packag. Manuf. Technol..

[12] Hedlund C., Blom H.O., Berg S. (1994). Microloading effect in reactive ion etching. J. Vac. Sci. Technol. A.

[13] Gerlt M.S., Läubli N.F., Manser M., Nelson B.J., Dual J. (2021). Reduced etch lag and high aspect ratios by deep reactive ion etching (DRIE). Macromachines.

[14] Choi J.W., Guan O.L., Yingjun M., Yusoff H.B.M., Jielin X., Choi Lan C., Leng Loh W., Long Lau B., Hwee Hong L., Guan Kian L. (2014). TSV Cu filling failure modes and mechanisms causing the failures. Trans. Compon. Packag. Manuf. Technol..

[15] Kim K.H., Kim S.C., Park K.Y., Yang S.S. (2011). DRIE fabrication of notch-free silicon structures using a novel silicon-on-patterned metal and glass wafer. J. Micromechan. Microengineering.

[16] Vandevelde B., Okoro C., Gonzalez M., Swinnen B., Eric B. Thermo-mechanics of 3D-wafer level and 3D stacked IC packaging technologies. Proceedings of the EuroSimE 2008-International Conference on Thermal. Mechanical and Multi-Physics Simulation and Experiments in Microelectronics and Micro-Systems.

[17] Chan J.M., Cheng X., Lee K.C., Kanert W., Sengtan C. Reliability evaluation of copper (Cu) through-silicon vias (TSV) barrier and dielectric liner by electrical characterization and physical failure analysis (PFA). Proceedings of the 2017 IEEE 67th Electronic Components and Technology Conference (ECTC).

[18] Kang S., Cho S., Yun K., Ji S., Bae K., Lee W., Kim E., Kim J., Cho J., Mun H. TSV optimization for BEOL interconnection in logic process. Proceedings of the 2011 IEEE International 3D Systems Integration Conference.

[19] Lall P., Bhat C., Hande M., More V., Vaidya R., Pandher R., Suhling J., Goebel K. Interrogation of system state for damage assessment in lead-free electronics subjected to thermo-mechanical loads. Proceedings of the 2008 58th Electronic Components and Technology Conference.

[20] Yuexing W., Linwei C., Xiangyu S., Quanfeng Z., Jie Z. (2022). Study of electromigration-induced void nucleation problem dominated by bulk, grain boundary and interfacial diffusion based on an improved energy approach. Packag. Manuf. Technol..

[21] Hau-Riege C., Klein R. The effect of a width transition on the electromigration reliability of Cu interconnects. Proceedings of the 2008 IEEE International Reliability Physics Symposium.

[22] Zhao J.H., Du Y., Morgen M., Ho P. (2000). Simultaneous measurement of Youngs modulus, Poisson ratio, and coefficient of thermal expansion of thin films on substrates. J. Appl. Phys..

[23] Engineering ToolBox (2003). Youngs Modulus, Tensile Strength and Yield Strength Values for some Materials [online]. https://www.engineeringtoolbox.com/young-modulus-d_417.html.

[24] Chang H.H., Shih Y.C., Hsu C.K., Hsiao Z.C., Chiang C.W., Chen Y.H., Chiang K.N. TSV process using bottom-up Cu electroplating and its reliability test. Proceedings of the 2008 2nd Electronics System-Integration Technology Conference.

[25] Ho S.W., Yoon S.W., Zhou Q., Pasad K., Kripesh V., Lau J. High RF performance TSV silicon kit for high frequency application. Proceedings of the 2008 58th Electronic Components and Technology Conference.

[26] Chiang C.H., Kuo L.M., Hu Y.C., Huang W.C., Co C.T., Chen K.N. (2013). Sealing bump with bottom-up Cu TSV plating fabrication in 3-D integration scheme. Electron. Device Lett..

[27] Yu A., Lau J.H., Ho S.W., Kumar A., Hnin W.Y., Lee W.S., Jong M.C., Sekhar V.N., Kripesh V., Pinjala D. (2011). Fabrication of high aspect ratio TSV and assembly with fine-pitch low-cost solder microbump for Si interposer technology with high-density interconnects. Trans. Compon. Packag. Manuf. Technol..

[28] Heryanto A., Putra W.N., Trigg A., Gao S., Kwon W.S., Che F.X., Ang X.F., Wei J., Made R.I., Gan C.L. (2012). Effect of copper TSV annealing on via protrusion for TSV wafer fabrication. J. Electron. Mater..

[29] Hsin Y.-C., Chen C.-C., Lau J.H., Tzeng P.-J., Shen S.-H., Hsu Y.-F., Chen S.-H., Wn C.-Y., Chen J.-C., Ku T.-K. Effects of etch rate on scallop of through-silicon vias (TSVs) in 200mm and 300mm wafers. Proceedings of the 2011 IEEE 61st Electronic Components and Technology Conference (ECTC).

